# Transcriptome analysis and molecular characterization of soluble chemical communication proteins in the parasitoid wasp *Anagrus nilaparvatae* (Hymenoptera: Mymaridae)

**DOI:** 10.1002/ece3.8661

**Published:** 2022-03-01

**Authors:** Ying Ma, Tingfa Huang, Bingjie Tang, Bingyang Wang, Liyang Wang, Jianbai Liu, Qiang Zhou

**Affiliations:** ^1^ State Key Laboratory of Biocontrol School of Life Sciences Sun Yat‐Sen University Guangzhou China; ^2^ School of Agriculture Sun Yat‐Sen University Guangzhou China; ^3^ Institute of Nanfan & Seed Industry Guangdong Academy of Sciences Guangzhou China

**Keywords:** *Anagrus nilaparvatae*, chemosensory proteins, Niemann‐pick type C2 proteins, odorant‐binding proteins, transcriptome

## Abstract

*Anagrus nilaparvatae* is an important egg parasitoid wasp of pests such as the rice planthopper. Based on the powerful olfactory system of sensing chemical information in nature, *A*. *nilaparvatae* shows complicated life activities and behaviors, such as feeding, mating, and hosting. We constructed a full‐length transcriptome library and used this to identify the characteristics of soluble chemical communication proteins. Through full‐length transcriptome sequencing, splicing, assembly, and data correction by Illumina, we obtained 163.59 Mb of transcriptome data and 501,179 items with annotation information. We then performed Gene Ontology (GO) functional classification of the transcriptome's unigenes. We analyzed the sequence characteristics of soluble chemical communication protein genes and identified eight genes: *AnilOBP2*, *AnilOBP9*, *AnilOBP23*, *AnilOBP56*, *AnilOBP83*, *AnilCSP5*, *AnilCSP6*, and *AnilNPC2*. After sequence alignment and conserved domain prediction, the eight proteins encoded by the eight genes above were found to be consistent with the typical characteristics of odorant‐binding proteins (OBPs), chemosensory proteins (CSPs), and Niemann‐pick type C2 proteins (NPC2s) in other insects. Phylogenetic tree analysis showed that the eight genes share low homology with other species of Hymenoptera. Quantitative real‐time polymerase chain reaction (RT‐qPCR) was used to analyze the expression responses of the eight genes in different sexes and upon stimulation by volatile organic compounds. The relative expression levels of *AnilOBP9*, *AnilOBP26*, *AnilOBP83*, *AnilCSP5*, and *AnilNPC2* in males were significantly higher than those in females, while the relative expression level of *AnilCSP6* was higher in females. The expression levels of *AnilOBP9* and *AnilCSP6* were significantly altered by the stimulation of β‐caryophyllene, suggesting that these two genes may be related to host detection. This study provides the first data for *A*. *nilaparvatae's* transcriptome and the molecular characteristics of soluble chemical communication proteins, as well as an opportunity for understanding how *A*. *nilaparvatae* behaviors are mediated via soluble chemical communication proteins.

## Graphical abstract

The structure predicted of 5 AnilOBPs and 2 AnilCSPs are spherical structures formed by α‐helices, and AnilNPC2 are spherical structures formed by β‐folded.

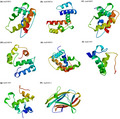

## 1 INTRODUCTION

The external environment of insects is complex and changeable. Volatile organic compounds in the environment can transmit information related to insect survival and reproduction, such as feeding, mating, and host foraging (Grosse‐Wilde et al., 2011; Stocker, 1994). Insects rely on their olfactory system to allow for the rapid and efficient use of chemical information in their environments (Leal, 2013; Turlings & Erb, 2018).

The insect olfactory system includes both the central and peripheral olfactory systems (Ong & Stopfer, 2012). When odor or pheromone molecules bind to olfactory or gustatory sensilla located on the insect cuticle, soluble chemical communication proteins transport these molecules to olfactory receptors on the peripheral nerve dendrites of sensory neurons (Benton et al., 2007). After the interaction of the signal molecules with the olfactory receptors, the chemical signals are converted into electrical ones that stimulate the dendritic nerves. The signals will ultimately be transmitted to the central nervous system to control the insect's behavior and physiological responses. The redundant odor or pheromone molecules will later be degraded by odorant‐degrading enzymes (ODEs) to restore the sensitivity of the sensory neuron (Jacquin‐Joly & Merlin, 2004; Leal, 2013; Zhou, 2010).

Soluble chemical communication proteins are the first participants in the olfactory system (Pelosi et al., 2006). They are mainly expressed in the peripheral lymph system responsible for the identification and transmission of odor molecules and pheromones that reach the olfactory receptors. Many soluble chemical communication proteins have been identified in insects, and their functions have been studied. Soluble chemical communication proteins include three major families: odorant‐binding proteins (OBPs), chemosensory proteins (CSPs), and Niemann‐pick type C2 proteins (NPC2s) (Pelosi et al., 2014). An OBP was first discovered in the antennae of male *Antheraea polyphemus*, and it was the first soluble binding protein identified in insects (Vogt & Riddiford, 1981). OBP functions include odor recognition, assistance in transporting odor molecules, and the degradation and removal of odor molecules (Kaissling, 1986; Krieger et al., 1996; Pelosi & Maida, 1995; Vogt et al., 1999). OBP expression was also found in insect gonads, eggs and feet (De Biasio et al., 2015), which are involved in development, reproduction, and stress resistance (Bruno et al., 2018; Pelosi et al., 2018). OBPs are small, spherical, water‐soluble proteins. All insect OBPs have highly conserved cysteines. Their connected disulfide bonds are main factors affecting and maintaining their protein structures. Based on the number of conserved cysteines, OBPs have five classes: Classical, Dimer, Minus‐C, Plus‐C, and Atypical OBPs (Cui et al., 2017; Fan et al., 2011; Qu et al., 2021). Insect CSPs were first identified in the antennae of *Drosophila melanogaster* (McKenna et al., 1994). They were thought to be carriers of odor molecules and chemicals, and as being capable of binding to chemical messages (Peng et al., 2017). CSPs and OBPs are similar in many ways. They are both expressed in a high concentration in antennae (McKenna et al., 1994; Vogt, 1987), and both participate in the process of olfactory recognition. Both are small, compact polypeptides mainly composed of α‐helical domains that define a hydrophobic binding cavity (Campanacci et al., 2003; Sandler et al., 2000; Tegoni et al., 2004). CSPs are smaller than OBPs (approximately 12 kDa). The conserved domain of the insect pheromone‐binding family A10/OS‐D consists of four conserved cysteine sites forming two disulfide bonds (Cys1‐Cys2, Cys3‐Cys4) and contains 5–6 α helices (Sanchez‐Gracia et al., 2009). CSPs are evolutionarily conserved compared to OBPs, and their high conservation may explain why there are fewer CSPs than OBPs (Pelosi et al., 2018; Wanner et al., 2004). CSPs in distantly related insects also tend to have 40–50% similar amino acid residues, compared with 10%–15% similar residues for OBPs (Pelosi et al., 2006). Therefore, it is assumed that CSPs are less specific for the selective binding of compounds, have a wider binding range, and have a more flexible binding ability (Wang et al., 2019). NPC2s in insects are similar to OBPs in functionality (Ishida et al., 2014; Pelosi et al., 2014; Zheng et al., 2018). In contrast to OBPs and CSPs, the secondary structure of NPC2 in insects is mainly β‐sheet based, which forms a larger internal binding cavity (Ishida et al., 2014). There are also conserved cysteines in the NPC2 sequence, which connect 2 or 3 disulfide bonds to maintain its stable three‐dimensional structure (Zhu et al., 2018).

*Anagrus nilaparvatae* (Pang et Wang) (Hymenoptera: Mymaridae) is the main egg parasite of the rice pest rice planthopper. *A*. *nilaparvatae* is widely used as a biological control agent in rice production (Zheng et al., 2017). To find hosts and supplemental food, these parasitoids need to receive and process information from rice plant volatile organic compounds and regulate its behavior to adapt to the environment. *A*. *nilaparvatae* can distinguish between the volatile organic compounds released by rice and can also use information from compounds produced by rice that have been consumed by the brown rice planthopper *Nilaparvata lugens* (Stål) to locate *N*. *lugens* eggs (Lou et al., 2005, 2006). *A*. *nilaparvatae* can also locate the eggs of the brown planthopper using rice volatile organic compounds such as (E)‐2 hexenal, methyl salicylate, caryophyllene, and linalool (Xiao et al., 2012). Some plant essential oils are also attractants of *A*. *nilaparvatae*, which help it locate and control pests (Mao et al., 2018). In addition, *A*. *nilaparvatae* can identify important wintertime habitat and food sources from vegetation volatile organic compounds in the field. For example, they can accurately locate *Impatiens balsamina*, *Emilia sonchifolia*, and *Sesamum indicum* to access essential food supplements providing increased longevity and parasitic efficiency (Zhu et al., 2013).

Although there have been previous studies on the effects of plant volatile organic compounds on the behavior of *A*. *nilaparvatae*, related to molecular biology research is only on mitochondrial COⅠ (cytochrome oxidase subunit Ⅰ) and 28s, 5.8s ribosomal genes. The molecular features have not been analyzed. The purpose of this study was to examine the characteristics of soluble chemical communication protein genes in *A*. *nilaparvatae* and help reveal the mechanisms involved in host detection and parasitism. We constructed a full‐length transcriptome library, obtained soluble chemical communication protein candidate genes, and analyzed the sequence characteristics of these genes. The expression patterns of these genes in both sexes, stimulated by volatile organic compounds, were quantitatively analyzed. This study provides a basis for the study of the molecular characteristics of the parasitoid and provides a reference for further revealing the molecular mechanisms behind its behavior.

## 2 MATERIALS AND METHODS

### 2.1 Insects

The hosts *N*. *lugens* were collected from rice paddy fields at the farm of the South China Agricultural University in Guangdong Province (N 23°9′3″, E 113°20′2″) in 2016 and reared with rice hydroponic seedlings. *A*. *nilaparvatae* were collected in the paddy field of the farm of South China Agricultural University in 2018 and were stably cultured for 60 generations on rice seedlings with the eggs of *N*. *lugens* in an insect cage (120 mesh gauze). The insect cage was placed in an insect incubator (GXZ‐380D, Ningbo Jiangnan Instrument Factory, Zhejiang, China), and the rearing conditions were as follows: 14:10 h (L:D) photoperiod, 25°C temperature, and 80% humidity.

### 2.2 RNA extraction

Each replicate pooled of 150 male and 150 female adult bodies, and three replicates were performed. Total RNA was extracted using TRIzol reagents (Eastep^®^ Super reagents, Promega, Shanghai, China) according to manufacturer instructions, and DNase Ⅰ in the reagents were used to remove contaminating genomic DNA. RNA concentration and purity were detected by Nanodrop 2000c (Thermo Fisher, Waltham, MA, USA), and sample integrity was detected by an Agilent 2100 (Hewlett‐Packard, Shanghai, China) (Rin value *d* > 7, RNA > 2 µg).

### 2.3 Transcriptome library construction, sequencing, and functional annotation

Transcriptome sequencing and library construction were performed by the Tiangen Biochemical Technology Company (Beijing, China).

For second‐generation sequencing, mRNA was enriched by magnetic beads with Oligo (dT) and broken into short fragments by fragmentation buffer under high temperature. Using mRNA as a template, the first cDNA strand was synthesized by adding six base random primers, and then, a second cDNA strand was synthesized. The ends of the double‐stranded cDNA were repaired and polyA was added to the 3’ ends. The cDNA fragments with connectors were enriched by polymerase chain reaction (PCR) amplification and sequenced on a HiSeq^TM^ 4000 platform (Illumina, San Diego, CA, USA). The process of third‐generation sequencing was conducted according to the standard protocol provided by Oxford Nanopore Technologies (ONT) (Jain et al., 2016). RNA was reverse‐transcribed into cDNA, and a switch oligo was added. Then, each RNA strand was digested and a second strand was synthesized. Repaired and purified DNA was then sequenced on the machine PromethION 48 (ONT Ltd., Oxford, UK).

Full‐length reads were obtained after we used Pychopper to filter short fragments and low‐quality reads and remove joints of raw fastq data from Nanopore sequencing. Then, ONclust2 software was used to cluster and correct the consensus sequences obtained from the reads. Finally, CD‐HIT was used to cluster the full‐length transcripts and remove the redundant sequences with more than 90% similarity (Zhao et al., 2021). Raw image data files obtained by Illumina sequencing were transformed into original Sequenced Reads/Raw Data by Base Calling analysis. TrimMomati software was used to remove the joint sequence of reads. After filtering the second‐generation short sequence data, we compared them to the obtained full‐length transcript sequence using BWA software and then sorted the comparison results. The full‐length transcript was corrected by Pilon according to the comparison results of the second‐generation data.

Transdecoder software was used to predict potential coding sequences (CDS). To obtain comprehensive gene function information, six major databases were annotated, including Pfam (protein family), Uni‐prot (universal protein), NR (NCBI nonredundant protein sequences), NT (NCBI nucleotide sequences), GO (Gene Ontology), KEGG (Kyoto Encyclopedia of genes and genomes), and TF (transcription factor).

### 2.4 Retrieval and structural analysis of soluble chemical communication protein genes

After annotating the amino acid sequence of unigenes, the soluble chemical communication protein genes were obtained from the annotation of NR, KEGG, and Uni‐prot databases. The obtained sequences were compared in NCBI BLAST (https://blast.ncbi.nlm.nih.gov/Blast.cgi). The open reading frame (ORF) and the amino acid sequence of the proteins expressed by these genes were predicted by the NCBI ORF Finder (https://www.ncbi.nlm.nih.gov/orffinder/). The molecular size and isoelectric point of the protein were predicted by the ProtParam tool (https://web.expasy.org/protparam/). SignalP (http://www.cbs.dtu.dk/services/SignalP/) was used to predict the signal peptide of those proteins. SWISS‐MODEL (https://swissmodel.expasy.org/interactive) was used to predict the three‐dimensional structure of the proteins, and Pfam (http://pfam.xfam.org/) was used to search for the conserved domain of protein sequences.

### 2.5 Phylogenetic analysis

Homologous sequences of obtained genes were retrieved using the online tool BLAST. Phylogenetic trees were constructed by the neighbor‐joining method, as implemented by MEGA 7.0 software, in combination with soluble chemical communication proteins from the published database of Hymenoptera species. Node support was assessed using a bootstrap procedure with 1000 replicates (Tamura et al., 2013).

### 2.6 RT‐qPCR

β‐caryophyllene is a volatile organic compound chemical that can attract *A*. *nilaparvatae* (Lou et al., 2005). It was used as a stimulus to compare changes in the expression levels of eight soluble chemical communication proteins. Samples of three groups were tested: (1) untreated female wasps; (2) female wasps stimulated by 0.01 g/L β‐caryophyllene for 1 h; (3) untreated male wasps. All wasps were healthy and unmated, and each sample contained 100 wasps, with 3 replicates per group. After RNA extraction, the kit GoScript^TM^ Reverse Transcription System (Promega, Madison, WI, USA) was used for reverse transcription PCR on a Veriti 96 Well Thermal Cycler (Gene Company Ltd., Hong Kong, China), and the kit Gotaq^®^ qPCR Master Mix A6002 (Promega, Madison, WI, USA) was used for quantitative PCR on a Light Cycler 480 (Roche, Shanghai, China). The temperature‐cycling parameters were as follows: 25°C for 5 min, 42°C for 75 min, 70°C for 15 min, and then 4°C until the end of the study. The procedure for quantitative PCR was as follows: Pre‐deformation took place at 95°C for 30 s, followed by 40 cycles of 95°C for 5 s, 53°C for 15 s, and 72°C for 20 s. The melting curve was performed at 65°C, 15 s. After the reaction, Light CyCler480 software was used to analyze the real‐time PCR amplification and melting curves, and the relative expression of the target gene was analyzed according to the 2^−△△Ct^ method. The primer design used Primer3 (v.0.4.0) (https://bioinfo.ut.ee/primer3‐0.4.0/). The primer sequences and amplicon length are shown in Table S1.

### 2.7 Statistical methods

Data are expressed as the mean SE of at least three biological replicates. SPSS 18.0 software (SPSS Inc.) was used for statistical analysis. The differences in levels of expression of the eight soluble chemical communication protein genes in response to β‐caryophyllene stimulation and the differences in levels of expression between male and female wasps were determined by *t* tests. Data are presented as the mean of three replicates (*n* = 3) ± SE. Different lower cases indicate significant differences (*p* < .05).

## 3 RESULTS

### 3.1 Transcriptome analysis

A total of 163.59 Mb of data was obtained after clustering and correcting the raw data obtained from nanopore transcriptome sequencing. A total of 224,251 unigenes were obtained. The longest sequence was 11,203 bp, the average length was 729.27 bp, and the N50 was 998 bp. After Illumina correction, the longest sequence was 11,225 bp, with an average length of 729.49 bp, and an N50 of 998 bp. The statistical results of the nanopore sequencing data and the data corrected by Illumina are shown in Table S2. Among the unigenes, 151,454 (67.79%) were between 200 and 700 bp in length, and 46,207 (20.68%) were more than 1000 bp long. Owing to full‐length transcriptome sequencing, the sequencing was complete. After multiple corrections, the quality of the data group was increased. The original transcriptome data results and length distribution statistics are shown in Table 1.

**TABLE 1:** Statistical results of transcriptome sequencing of *Anagrus nilaparvatae*

Length range	Raw data	Polished
200–700	7,506,513 (72.24%)	151,454 (67.79%)
700–1200	1,438,724 (13.66%)	37,465 (16.52%)
1200–1700	705,180 (6.7%)	17,363 (7.66%)
1700–2200	429,750 (4.08%)	9262 (4.08%)
2200–2700	169,499 (1.61%)	4491 (1.98%)
2700+	141,574 (1.36%)	3369 (1.51%)
Number of contig	10,405,444	224,251
Large contig(≥1000bp)	1,882,079	46,207
N50 length(bp)	880	998
Mean contig length(bp)	695.59	729.49

Note

Length range: The Length range of the transcript; Raw data: Nanopore transcriptome sequencing data; Polished: Unigenes obtained from the transcriptome sequencing data after being corrected by Illumina; Number of contig: Number of effective reads; Large contig(≥1000 bp): Number of reads longer than 1000 bp; N50 Length (bp): Sent the obtained unigene in order of length from large to small, and successively add up the length of unigenes until the length is no less than 50% of the total length; Mean Contig Length (bp): Mean length of unigenes.

### 3.2 Functional annotation of unigenes

All the unigene sequences were compared in the NT, NR, Uni‐prot, GO, KEGG, Pfam, and TF databases. The results showed database annotation information in the NCBI official nucleic acid database, protein database, studied protein database, GO functional classification, KEGG metabolic pathway, protein family database, NCBI protein database, and the transcription factor database. A total of 501,179 items of annotation information were obtained from the transcriptome of the adult wasps, and the annotation information matched 76,326 sequences in the database (Table 2).

**TABLE 2:** Unigenes annotated in different databases

Database	Query number	Percentage	Target number
KEGG	73,529	32.79%	10,177
NR	82,111	36.62%	20,983
NT	142,990	63.76%	12,157
Pfam	22,482	10.03%	3040
TF	6324	2.82%	192
Uni‐prot	84,735	37.79%	17,760
GO	89,008	39.69%	12,017

Note

Database: Name of Database; Query number: Unigenes number; Percentage: Percentage of unigenes compared to the database; Target number: Number of sequences match in the database.

**FIGURE 1:**
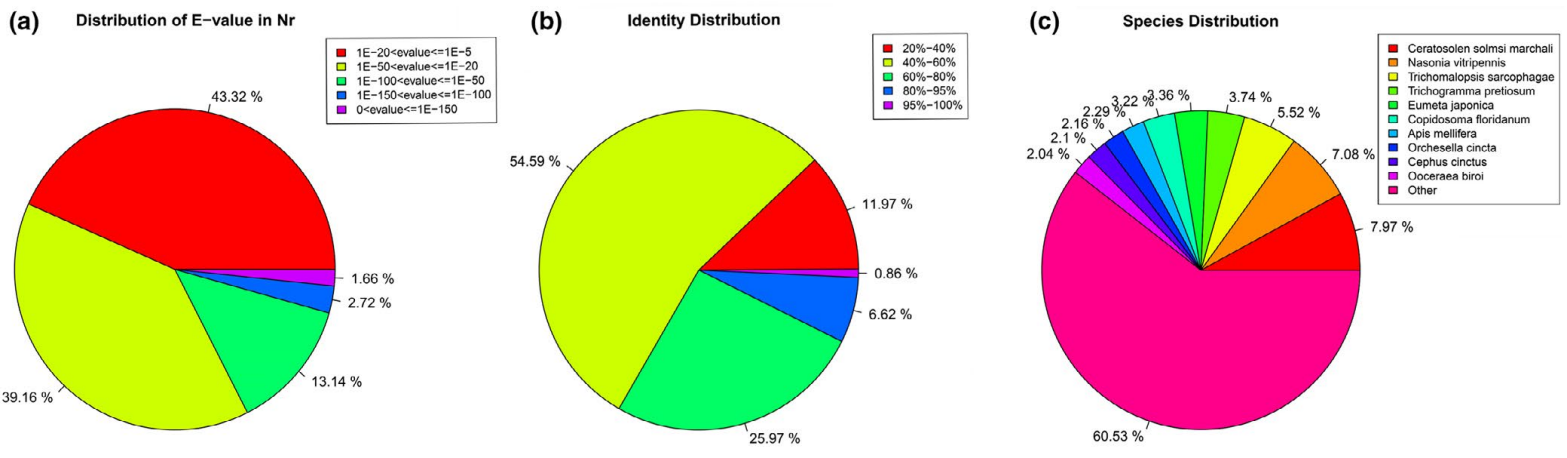
*Anagrus nilaparvatae* unigenes classification in the NR database. (a) E‐value distribution of NR annotation results. (b) Identity distribution of the NR annotation results. (c) Species distribution of the NR annotation results

Among the above database comparison results, the NR database had the most identical sequences, so the NR database could better cover the output results and fully analyze the sequence homology. According to the distribution statistics of the data with E‐value <1.0E−5, 17.5% (0–1E−50) alignment sequences showed extremely strong homology, 39.16% (1E‐50–1E−20) alignment sequences showed strong homology, and the remaining 43.32% (1E‐20–1E−5) sequences showed moderate homology (Figure 1a). Among the sequence alignments, the similarity between 7.48% of the sequences and the NR database was higher than 80%, 25.97% of the sequences had similarities between 60% and 80%, and 54.59% of the sequences had less than 60% similarity (Figure 1b). In the comparison results based on NR data, *Ceratosolen solmsi* Marchali (60.53%) had the largest number of matches, followed by *Nasonia vitripennis* (7.08%), *Trichomalopsis sarcophagae* (5.52%), and *Trichogramma pretiosum* (3.74%) (Figure 1c).

**FIGURE 2:**
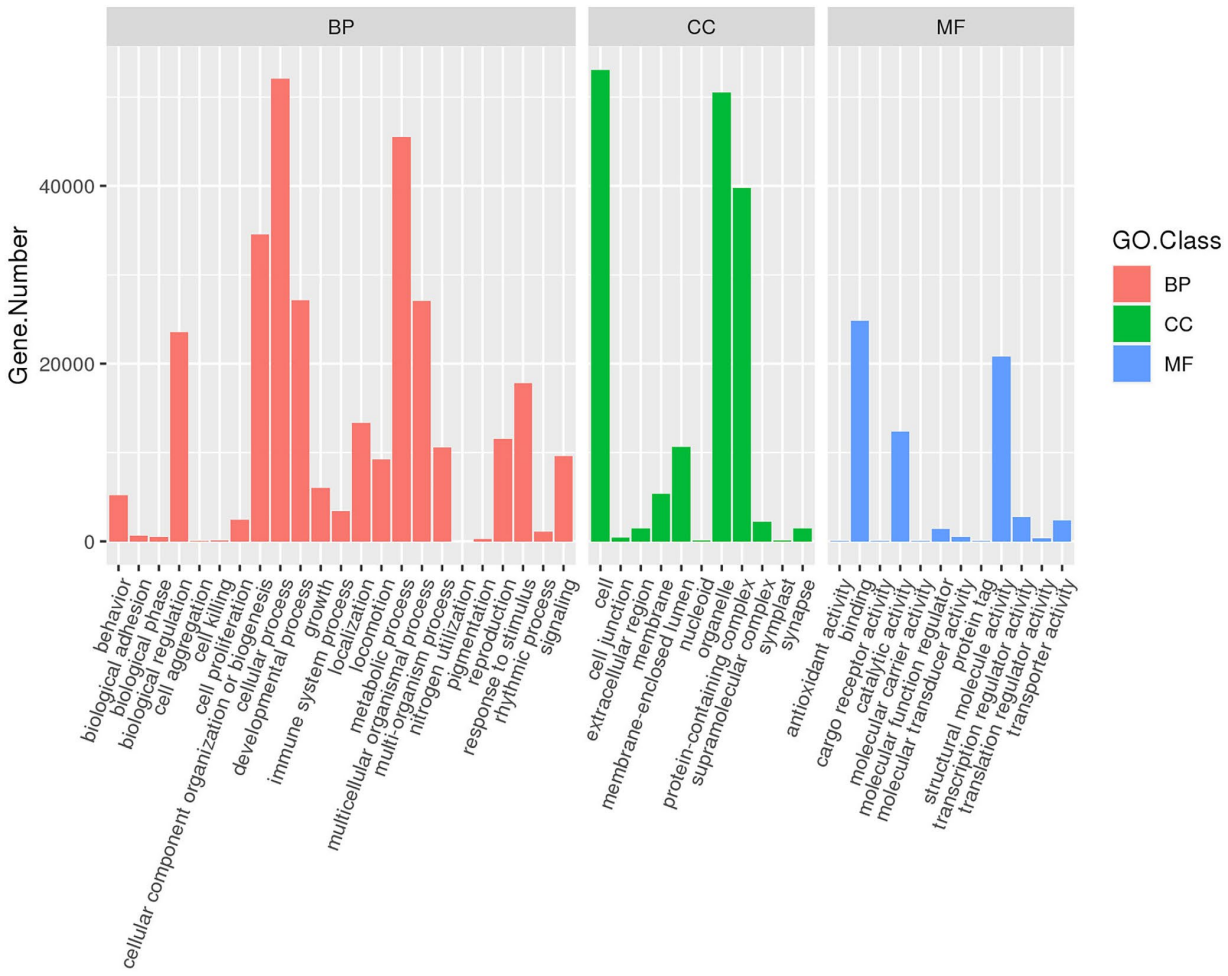
GO functional classification of *Anagrus nilaparvatae* transcriptome unigenes. The horizontal coordination, the GO function annotated to the second hierarchical classification; the vertical coordination, the number of genes annotated to the GO classification. Annotations belonging to different GO macrocategories are separated from each other and represented by different colors; BP means biological process, CC means cellular component, MF means molecular function

A total of 89008 (39.69%) unigenes from the transcriptome were annotated into the GO database. Among all the unigenes, 303,636 correspond to biological processes, 166,144 correspond to cellular components, and 65,857 correspond to molecular functions. These three categories were further divided into 45 secondary functional annotations, among which biological processes were divided into 22 secondary functional items, cellular components into 11 secondary functional items, and molecular functions into 12 secondary functional items. In biological processes, the greatest number of unigenes were commented to the cellular process (52,544), followed by metabolic processes (45,901) and cellular component organizations or biogenesis (34,715). Among the cellular components, the greatest number of unigenes was annotated to cell (53,422) and organelle (50,859). Among molecular functions, binding (24,952) and structural molecule activity (20,805) had the most notes (Figure 2).

### 3.3 Identification and bioinformatics analysis of soluble chemical communication protein genes

In the transcriptome, 21 OBPs, 5 CSPs, and 2 NPCs were found in Pfam; 10 OBPs and 1 CSP were found in NR; 18 OBPs and 1 NPC were found in Uni‐Prot; and 15 OBPs, 1 CSP, and 1 NPC2 were found in KEGG.

For the retrieved sequences, 34 OBPs, 5 CSPs, and 2 NPCs were left after unigenes with the same sequence number were removed. Then, MEGA 7.0 software was used to compare nucleic acid similarity and eliminate the repeated unigenes, leaving 5 OBPs, 2 CSPs, and 1 NPC2. ORF Finder was then used to obtain the ORF of the sequence and the protein sequence, Pfam was used to retrieve the protein sequence domain, and BLAST was used to retrieve the homologous proteins to name the gene (Table 3). The transcriptome sequencing found 5 OBPs, 2 CSPs, and 1 NPC2, namely, *AnilOBP2*, *AnilOBP9*, *AnilOBP26*, *AnilOBP56*, *AnilOBP83a*, *AnilCSP5*, *AnilCSP6*, and *AnilNPC2*, respectively, corresponding to predicted proteins for AnilOBP2, AnilOBP9 AnilOBP26, AnilOBP56, AnilOBP83a, AnilCSP5, AnilCSP6, and AnilNPC2. Those sequences with orthologous sequences can be found in Figure S1.

**TABLE 3:** List of olfactory binding protein genes alignment of *Anagrus nilaparvatae*

Gene name	Homology search with known protein				
	Scientific name	E‐value	Identity	Score	Protein ID
*AnilOBP2*	*Trichogramma dendrolimi*	6E−73	78.63%	225	ANG08492.1
*AnilOBP9*	*Apis mellifera*	0.001	26.61%	47	NP_001035315.1
*AnilOBP26*	*Nasonia vitripennis*	2.20E−20	43.91%	105.1	G8B1N1_NASVI
*AnilOBP56*	*Nylanderia fulva*	2E−28	42.86%	111	XP_029157120.1
*AnilOBP83*	*Nasonia vitripennis*	1E−32	45.63%	121	XP_016842824.1
*AnilCSP5*	*Trichogramma dendrolimi*	2E−20	69.49%	88.6	ANG08519.1
*AnilCSP6*	*Apis mellifera*	4.10E−09	30.68%	66.6	NP_001071287
*AnilNPC2*	*Trichogramma pretiosum*	9E−45	52.32%	154	XP_014228936.1

### 3.4 Sequence characterization of soluble chemical communication protein genes

After ORF prediction of the partial nucleic acid sequence, the molecular weight, isoelectric point, hydrophilicity, and signal peptide of the proteins were predicted and are shown in Table 4.

**TABLE 4:** Characteristic of eight olfactory binding proteins of *Anagrus nilaparvatae*

Gene name	ORF length (nt)	Protein length (aa)	Molecular weight (Da)	pI	Grand average of hydropathicity (GRAVY)	Signal peptide
*AnilOBP2*	441	146	16542.92	4.74	−0.049	N′(19aa)
*AnilOBP9*	431	132	15272.52	5.64	−0.573	N′(21aa)
*AnilOBP26*	420	139	15859.42	8.96	0.631	N′(16aa)
*AnilOBP56*	396	131	13948.57	8.17	0.037	N′(16aa)
*AnilOBP83*	312	103	11556.32	4.78	−0.206	–
*AnilCSP5*	234	77	8760.29	9.05	−0.375	N′(29aa)
*AnilCSP6*	483	160	18896.61	9.61	−0.838	–
*AnilNPC2*	465	154	17070.87	8.51	0.185	N′(19aa)

**FIGURE 3:**
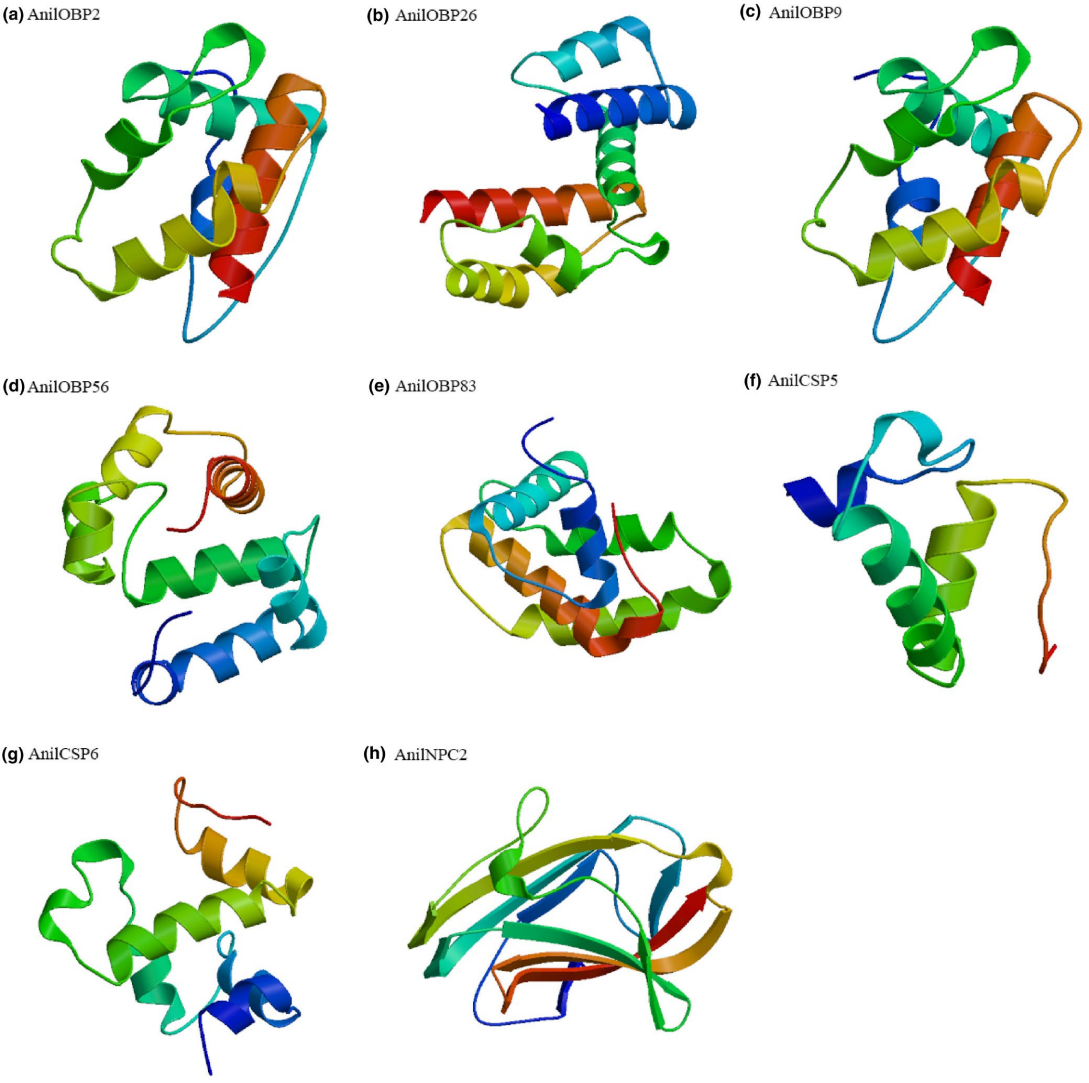
Three‐dimensional structure prediction of soluble chemical communication proteins in *Anagrus nilaparvatae*. (a–e) Structure prediction of AnilOBPs. (f, g) Structure prediction of AnilCSPs. (h) Structure prediction of AnilNPC2

The ORFs of the five AnilOBPs had a range of 312 to 441 bases encoding 103 to 146 amino acids with molecular weights ranging from 11.56 to 16.54 kD. AnilOBP26 and AnilOBP56 are hydrophobic proteins with acidic isoelectric points, while AnilOBP2, AnilOBP9, and AnilOBP83 are hydrophilic proteins with alkaline isoelectric points. Except for AnilOBP83, the other four OBPs have signal peptide sequences at the N terminal. The conserved domain of OBPs, namely, the GOBP family, has six conserved cysteines, and these six cysteines form three disulfide bonds. The bond formation rules are Cys1‐Cys4, Cys2‐Cys5, and Cys3‐Cys6. All of the six predicted amino acid AnilOBP sequences, except for AnilOBP83, had intact conserved domains. Specifically, the cysteine spacing pattern between amino acids 24 and 138 of AnilOBP2 is C1‐X26‐C2‐X3‐C3‐X39‐C4‐X12‐C5‐X8‐C6 (X means any amino acid). The cysteine spacing pattern of AnilOBP9 was C1‐X29‐C2‐X3‐C3‐X41‐C4‐C11‐X8‐C6 between amino acids at amino acid sites 22 to 130. The cysteine spacing pattern of AnilOBP26 was C1‐X27‐C2‐X3‐C3‐X39‐C4‐X8‐C5‐X8‐C6 between amino acid sites 19 and 131. The cysteine spacing pattern of AnilOBP56 was C1‐X27‐C2‐X3‐C3‐X38‐C4‐X8‐C5‐X8‐C6 between amino acid positions 15 and 125. The cysteine spacing pattern of AnilOBP83 was C1‐X1‐C2‐X1‐C3‐X41‐C4‐X9‐C5‐X8‐C6 between amino acid positions 1 and 95. The predicted three‐dimensional structures of the five AnilOBPs proteins were mainly composed of α helix structures (Figure 3a–e).

The ORF of *AnilCSP5* and *AnilCSP6* have 234 and 483 bases, each encoding 77 and 160 amino acids with molecular weights of 8.76 kD and 18.89 kD, respectively. The isoelectric points of AnilCSP5 and AnilCSP6 are alkalic and hydrophilic proteins. There are 29 amino acid residues at the N terminal of AnilCSP5, which are signal peptide sequences, while AnilCSP6 is not annotated to a signal peptide. The conserved domains of two AnilCSPs were predicted by Pfam. The conserved domains of AnilCSP5 were between amino acids 4 and 63, but were not complete. The conserved domain of AnilCSP6 was located between amino acids 26 and 115, and the cysteine spacing pattern was C1‐X6‐C2‐X18‐C3‐X2‐C4. The predicted three‐dimensional structures of the two AnilCSPs were mainly composed of α helix structures (Figure 3f, g).

The ORF of *AnilNPC2* has 465 bases and encodes 154 amino acids, and the molecular weight of AnilNPC2 is 17.07 kD. The isoelectric point of AnilNPC2 is 8.51, and it is a hydrophobic protein. The conserved domain prediction results showed that it belongs to the lipid binding protein family and possesses the ML domain (MD‐2‐related lipid recognition domain), which is located between amino acids 19 and 153 and has six conserved cysteines. The cysteine spacing pattern was C1‐X15‐C2‐X4‐C3‐X46‐C4‐X12‐C5‐X39‐C6. The three‐dimensional structure of AnilNPC2 protein is mainly composed of β folds (Figure 3h).

### 3.5 Phylogenetic analysis of soluble chemical communication proteins

**FIGURE 4:**
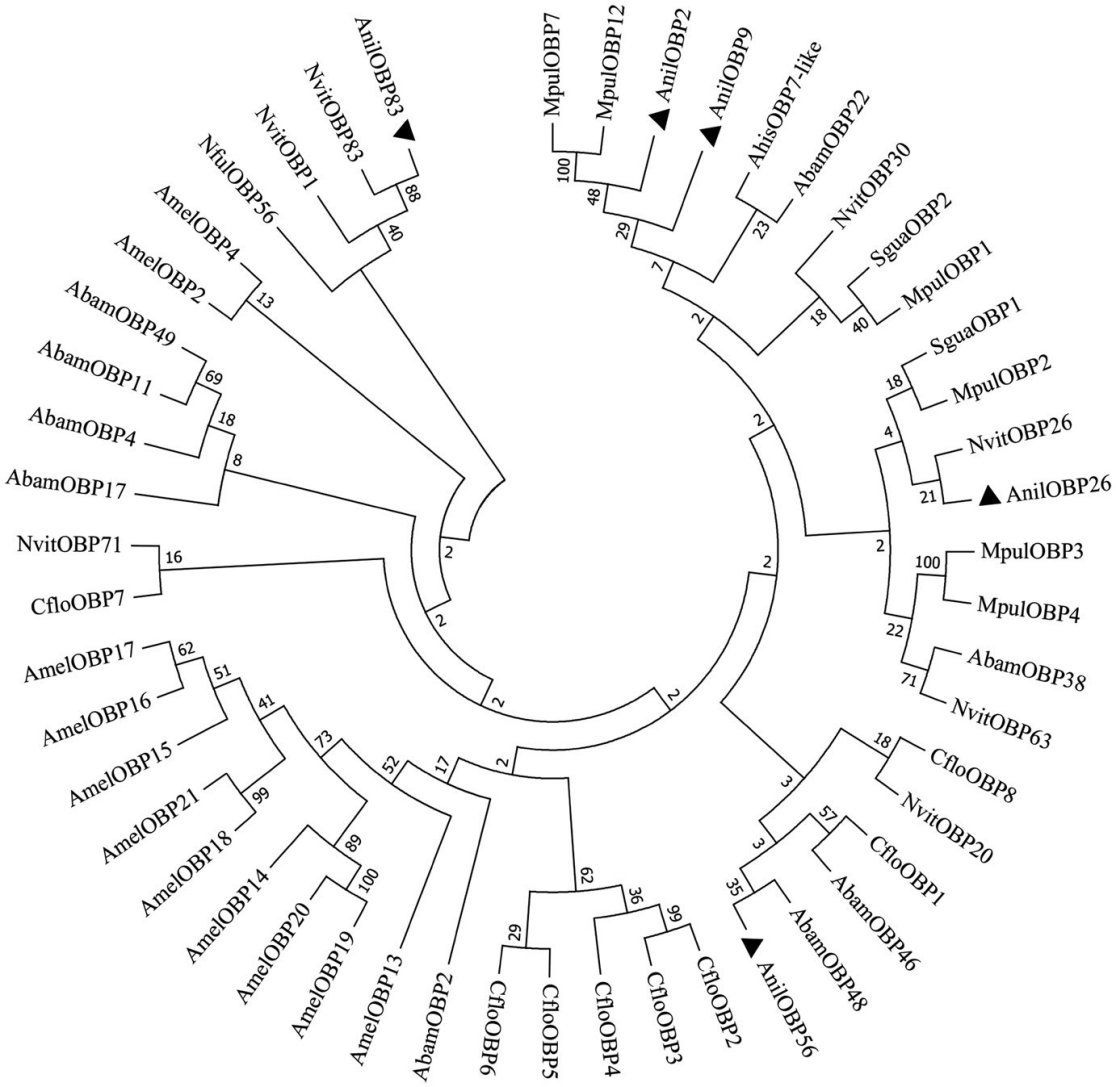
Neighbor‐joining tree of AnilOBPs of *Anagrus nilaparvatae*. ▲ indicates *A*. *nilaparvatae* protein *AnilOBPs*, Sgua, *Sclerodermus guani*; Cflo, *Copidosoma floridanum*; Ahis, *Asecodes hispinarum*; Mpul, *Meteorus pulchricornis*; Abam, *Aenasius bambawalei*; Amel, *Apis mellifera*; Nvit, *Nasonia vitripennis*; Nful, *Nylanderia fulva*

**FIGURE 5:**
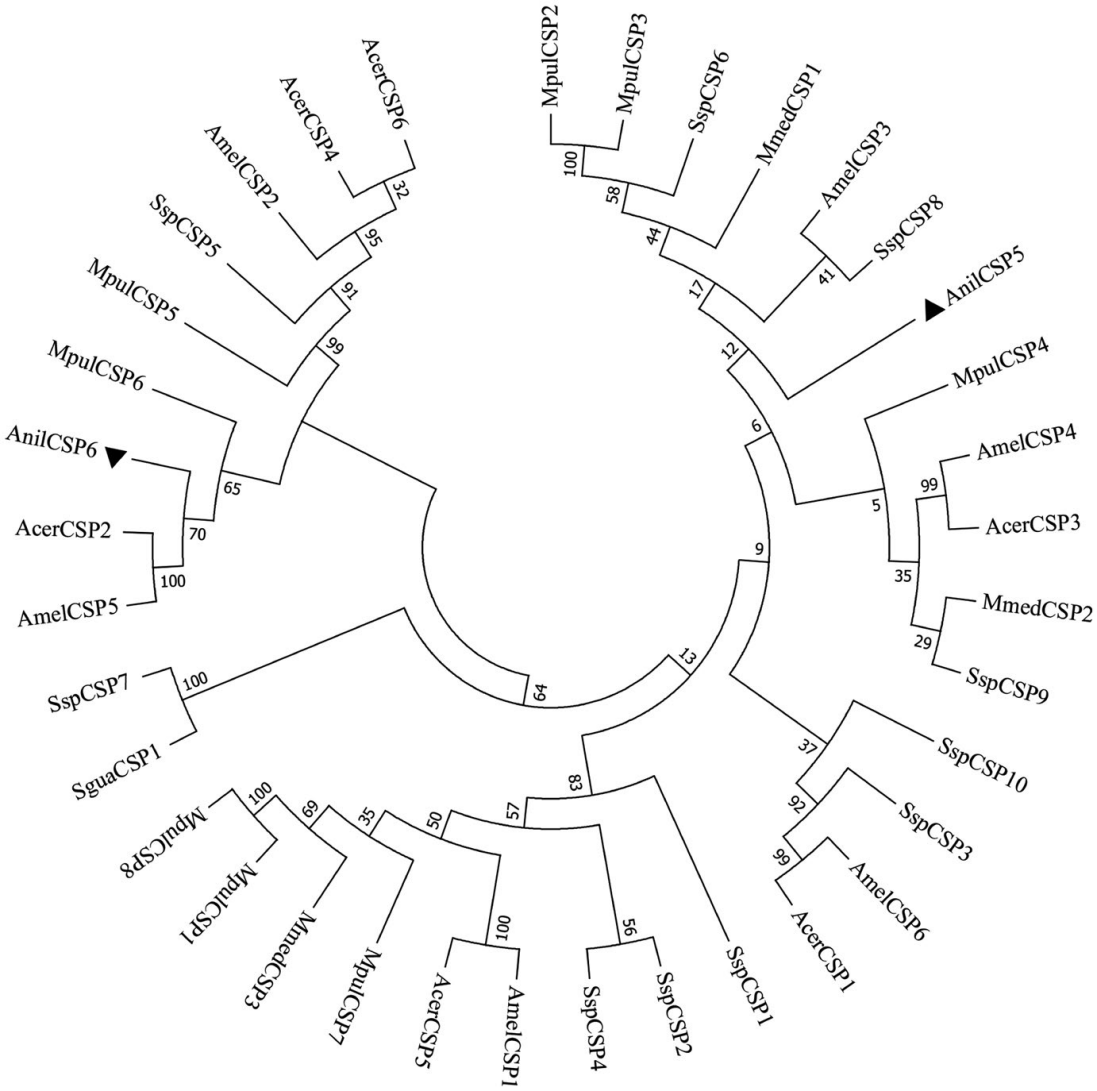
Neighbor‐joining tree of AnilCSPs of *Anagrus nilaparvatae*. ▲ indicates *A*. *nilaparvatae* protein *AnilCSPs*, Acer, *Apis cerana*; Amel, *Apis mellifera*; Mmed, *Microplitis mediator*; Ssp, *Sclerodermus sp*; Sgua, *Sclerodermus guani*; Mpul, *Meteorus pulchricornis*

**FIGURE 6:**
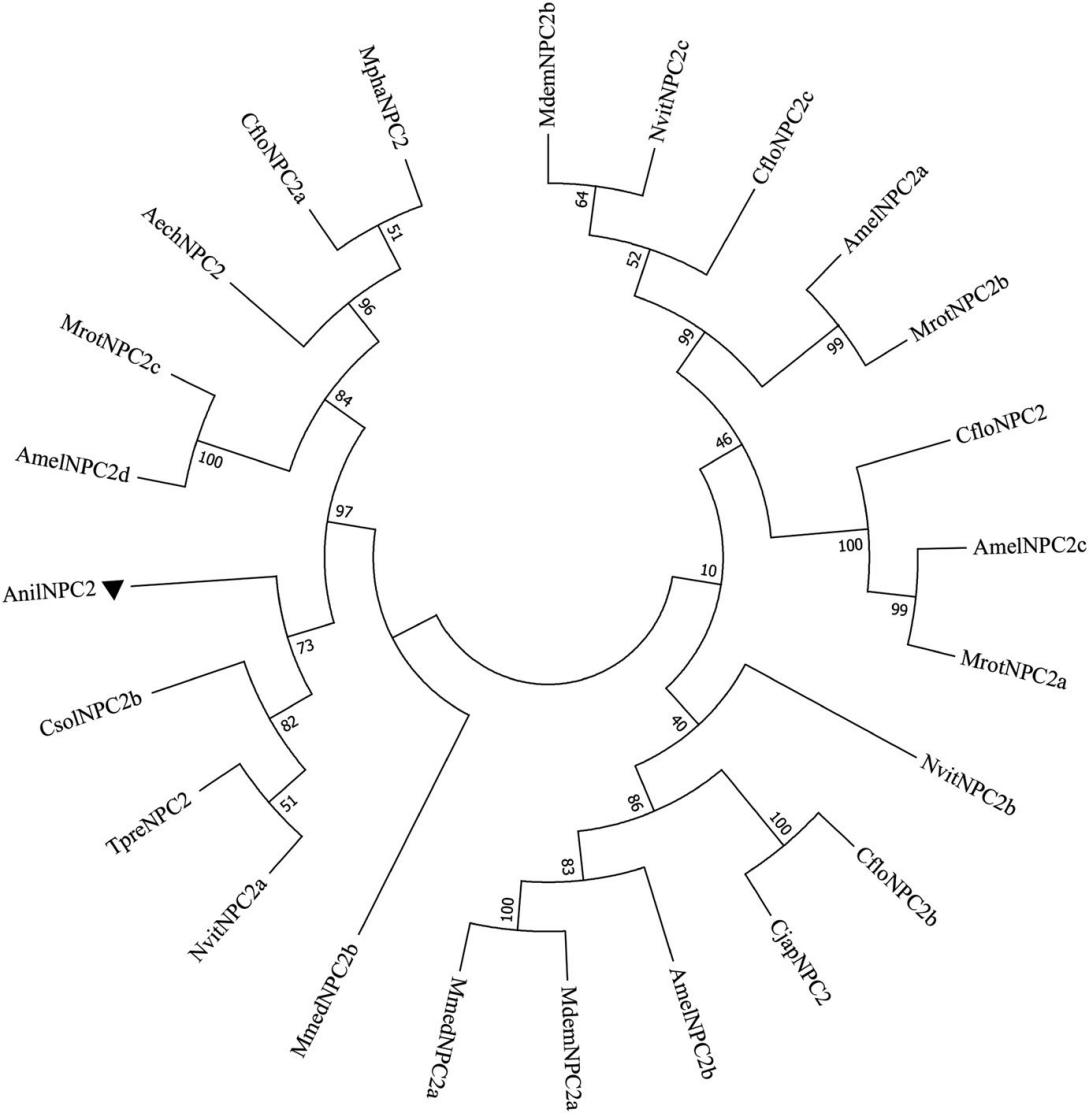
Neighbor‐joining tree of AnilNPC2 of *Anagrus nilaparvatae*. ▲ indicates *A*. *nilaparvatae* protein *AnilNPC2*, Amel, *Apis mellifera*; Aech, *Acromyrmex echinatior*; Cflo, *Copidosoma floridanum*; Cjap, *Camponotus japonicas*; Csol, *Ceratosolen solmsi* marchali; Mdem, *Microplitis demolitor*; Mmed, *Microplitis mediator*; Mrot, *Megachile rotundata*; Mpha, *Monomorium pharaonis*; Nvit, *Nasonia vitripennis*; Tpre, *Trichogramma pretiosum*

Three evolutionary trees, the OBPs of 9 species (Figure 4), the CSPs of 9 species (Figure 5) and the NPC2s of 11 species (Figure 6) were constructed using the neighbor‐joining (NJ) method. AnilOBP2 in *A*. *nilaparvatae* forms a clade with MpulOBP7 and MuplOBP12 (*Meteorus pulchricornis*). A clade with AnilOBP9; AnilOBP83 with NvitOBP83 (*N*. *vitripennis*), AnilOBP26 with NvitOBP26 and AnilOBP56 with AbamOBP48 (*Aenasius bambawalei*) were merged into one branch. The homology between AnilOBP2 and AnilOBP9 was found to be greater than that of other AnilOBPs. AnilCSP5 in *A*. *nilaparvatae* was found to be further away from the other CSPs. AnilCSP6 shared a clade with AmelCSP5 (*Apis mellifera*) and AcerCSP2 (*Apis cerana*), and another clade contained MpulCSP6 (*M*. *pulchricornis*) alone. AnilNPC2 in *A*. *nilaparvatae* forms a clade with CsolNPC2b (*C*. *solmsi*). TpreNPC2 (*T*. *pretiosum)* and NvitNPC2a (*N*. *vitripennis*) belong to the same clade, but AnilNPC2 is distant from the others.

### 3.6 Expression of soluble chemical communication protein genes response to β‐caryophyllene

**FIGURE 7:**
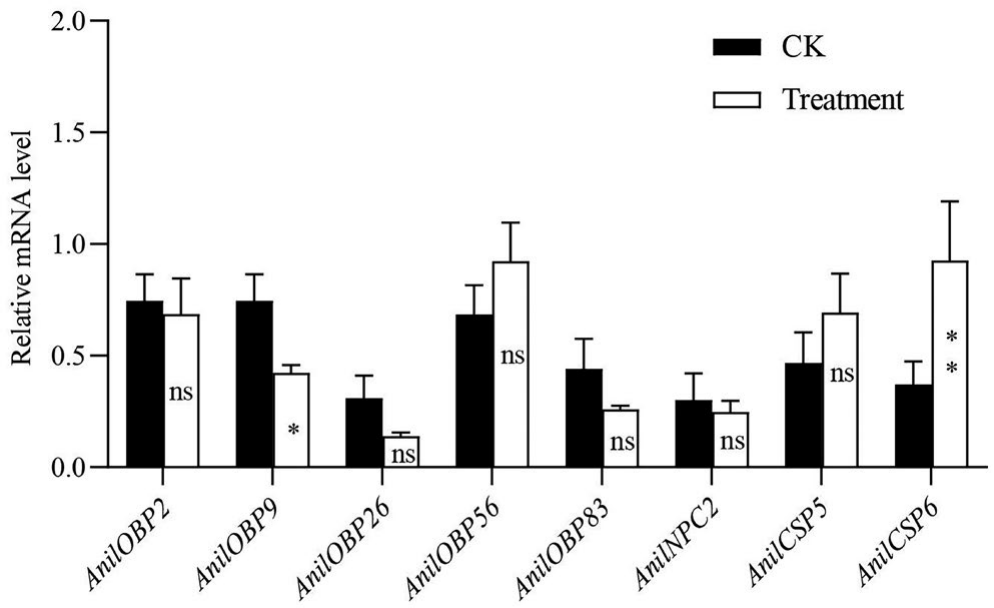
Expression profiles of soluble chemical communication protein genes in *Anagrus nilaparvatae* responding to β‐caryophyllene. Horizontal coordination, the soluble chemical communication protein genes of *A*. *nilaparvatae*. Vertical coordination, relative expression of different soluble chemical communication protein genes compared to *actin*. Results are presented as the mean ± SEM (*n* = 3). *^,^** indicates significant difference at the *p* < .05 and *p* < .01 level by Student's *t* test, respectively

After *A*. *nilaparvatae* was stimulated by β‐caryophyllene, the relative expression levels of *AnilOBP2*, *AnilOBP26*, *AnilOBP56*, *AnilOBP83*, *AnilCSP5*, and *AnilNPC2* were not significantly different, while *AnilOBP9* was significantly decreased and *AnilCSP6* was significantly increased (Figure 7).

### 3.7 Expression of soluble chemical communication protein genes in different sexes

**FIGURE 8:**
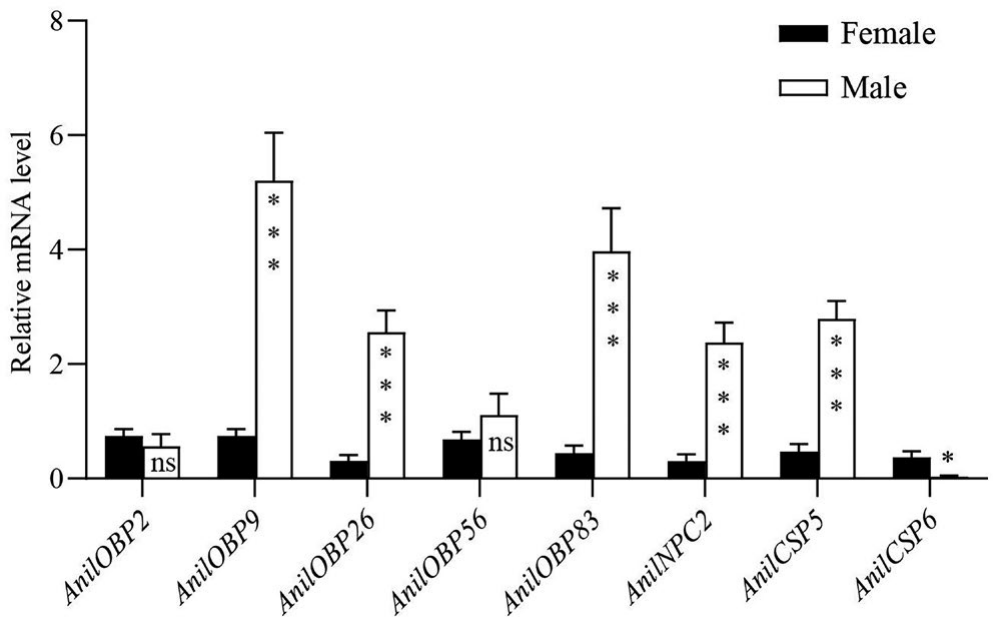
Expression profiles of soluble chemical communication protein genes in males and females of *Anagrus nilaparvatae*. Horizontal coordination, the soluble chemical communication protein genes of *A*. *nilaparvatae*. Vertical coordination, relative expression of different soluble chemical communication protein genes compared to *actin*. Results are presented as the mean ± SEM (*n* = 3). *^,^**^,^*** indicates significant difference at the *p* < .05, *p* < .01, *p* < .001 level by Student's *t* test, respectively

We quantified the differences in male and female wasp expression levels of the eight genes. There was no significant difference in the relative expression levels of *AnilOBP2* and *AnilOBP56* between males and females. The relative expression levels of *AnilOBP9*, *AnilOBP26*, *AnilOBP83*, *AnilCSP5*, and *AnilNPC2* in males were significantly higher than those in females. In contrast, the relative expression level of *AnilCSP6* in females was significantly higher in males (Figure 8).

## 4 DISCUSSION

We constructed a full‐length transcriptome database of *A*. *nilaparvatae*, an important natural enemy of the rice planthopper. A total of 10,405,444 reads with an average length of 695.59 bp were obtained by Nanopore sequencing. A total of 43,657,748 reads with an average quality of 35.88 bp were obtained by Illumina sequencing. Nanopore transcriptome sequencing technology produces more data and the read length is longer. This demonstrates the unique advantages of nanopore sequencing in identifying gene sequences. However, the nanopore sequencing platform has the disadvantage of having a high single base error rate. To improve base accuracy, the sequencing data of Illumina was combined with the corrected nanopore sequencing data, and 224,251 reads with an average length of 729.49 bp were finally obtained. This is the first time that third‐generation transcriptome sequencing has been used for a parasitic wasp.

By comparing the obtained unigenes with NT, NR, Uni‐Prot, and other public databases, 50,1179 items of annotation information were obtained. In the NR database, the sequence of *A*. *nilaparvatae* is highly similar to that of *C*. *solmsi*, but the transcription sequence annotation amount was low and the similarity degree of most sequences was lower than 60%. However, this provided a reference for the subsequent data mining of *A*. *nilaparvatae*. A large number of unigenes remain to be annotated in the full‐length transcripts. This could be for many reasons, such as the filtering threshold being too high or the database not identifying some proteins because they are not in the reference database. Furthermore, the annotation information of the transcriptome is derived from known insect genes, while the genomes of parasitoid wasps are not well studied (Branstetter et al., 2018).

Based on the annotated full‐length transcriptome data, eight soluble chemical communication protein genes were obtained by keyword retrieval and sequence alignment. These included five *OBP*s (*AnilOBP2*, *AnilOBP9*, *AnilOBP26*, *AnilOBP56*, *AnilOBP83*), two *CSP*s (*AnilCSP5*, *AnilCSP6*), and one *NPC2* (*AnilNPC2*). The transcripts annotated a small number of soluble chemical communication protein genes. Some reasons might be involved in this phenomenon, firstly, the filter threshold for eliminating redundant sequences maybe too high (90%). In addition, there has not been a previous report on the genome of *A*. *nilaparvatae*, and the database used to identify sequences may also be incomplete, so very little annotated information is available. Finally, using an antenna transcriptome may be more suitable for identifying chemosensory genes than a whole‐body transcriptome, so transcriptome of antennae or whole genome sequencing will be performed in the future for a more complete identification of chemosensory genes in this species. We found a low conservation of soluble chemical communication protein genes. For example, OBPs only shared 10%–15% of their residues between species, while CSPs often share 40%–50% identical residues between orthologues from phylogenetically distant species (Pelosi et al., 2006; Wang et al., 2019). The number of soluble chemical communication protein is highly variable among hymenopterans. For example, *Macrocentrus cingulum* has 3 OBPs (Ahmed et al., 2017), while *N*. *vitripennis* had 90 OBPs (Vieira et al., 2012); *Aphidius Ervi* had 2 CSPs (Ballesteros et al., 2017), while *Chouioia Cunea* had 11 CSPs (Zhao et al., 2016).

The structure and function of proteins correspond, so analyzing the structure of a protein can help predict its function. The structures of more than 20 OBPs have been determined by X‐ray crystallography and/or nuclear magnetic resonance (NMR) spectroscopy. Some were also complexed with ligands (Brito et al., 2016), and the structures of three CSPs are available (Jansen et al., 2007; Lartigue et al., 2002; Pelosi et al., 2018; Tomaselli et al., 2006). These are all spherical structures based on α‐helices. In contrast, the NPC2 structure of only one insect has been analyzed (*Camponotus japonicus*) (Ishida et al., 2014), and this structure is spherical and based on β‐sheets. The three‐dimensional structure of a protein can be predicted by software based on the amino acid sequence and used to analyze the spatial aspect of the protein and predict its possible function (Scieuzo et al., 2021). In this study, after sequence alignment and conserved domain prediction, eight proteins were identified as having typical characteristics of OBPs, CSPs, and NPC2s in insects. The predicted shapes of AnilOBPs and AnilCSPs are spherical structures formed by α‐helices, and AnilNPC2 is a spherical structure formed by β‐sheets. This is similar to the shape of CjapNPC2 in *C*. *japonicus* (Ishida et al., 2014). With the exception of AnilOBP83 and AnilCSP6, the other six proteins contained N‐terminal signal peptides, which may have the function of information binding and transport. The absence of AnilOBP83 and AnilCSP6 signal peptides may be related to incomplete sequencing.

Phylogenetic analysis is helpful to discover the evolutionary relationships of proteins and analyze the homology of species. By evolutionary tree analysis, we found the evolutionary distance of soluble chemical communication protein genes is far to each other in *A*. *nilaparvatae*. For previous similar works performed on parasitoids, it can be seen that in general, the OBPs are grouped into clades integrated of sequences from different species (Pelosi et al., 2006; Wang et al., 2019). Only in some cases, there is a clade expansion, such as *N*. *vitripennis* in which a clade composed only for OBP sequences of the same species, because it has a large OBP family (Vieira et al., 2012). *A*. *nilaparvatae* is similar to other models already studied due to the low number of identified sequences.

The study of expression profiles for soluble chemical communication protein genes is helpful for understanding of olfactory system in parasitic wasps at the molecular level. In the preliminary experiment, we used other reference genes, such as *gadph*, and finally, we selected the optimal one *actin* as the reference gene. All primers used in the study were pre‐tested in preliminary assays. The average amplification efficiency was between 0.8 and 1.0, determined by the 2^−△△Ct^ method (Livak & Schmittgen, 2001). β‐caryophyllene is a volatile organic compound released by rice to attract *A*. *nilaparvatae* searching for eggs of the rice planthopper (Lou et al., 2005). Behavioral experiments have also shown that β‐caryophyllene is attractive to rice planthopper parasitoid wasps (Xiao et al., 2012). The expression levels of *AnilOBP9* and *AnilCSP6* in females increased significantly in response to β‐caryophyllene stimulation. The expression of *AnilOBP9* decreased significantly and the expression of *AnilCSP6* increased significantly. Phylogenetic analysis showed that *AnilOBP9* has substantial homology with MpulOBP7 and MuplOBP12 (*M*. *pulchricornis*), both of which were highly expressed in antennae (Sheng et al., 2017). *AnilCSP6* has great homology with *AcerCSP2* (*A*. *cerana*), which was also highly expressed in antennae (Li et al., 2016). *AnilOBP9* and *AnilCSP6* may be related to olfactory perception and are involved in the sensing of β‐caryophyllene in *A*. *nilaparvatae*.

The expression of soluble chemical communication protein genes also showed sex differences. Except for *AnilOBP2* and *AnilOBP56*, the expression of other genes was different in males and females. Differences have also been reported for other insect species and are very common in parasitoid wasps. In *Rhodnius prolixus*, transcripts for *RproOBP17* and *RproOBP21* were enriched in female antennae and are possibly involved in the detection of oviposition attractants or other semiochemicals mediating female‐specific behaviors. *RproOBP26* and *RproOBP27* might be involved in the reception of sex pheromones, given that their transcripts were highly expressed in male antennae (Oliveira et al., 2018). *Locusta migratoria* males have many CSPs in their genitals; a total of 17 are abundantly expressed in the female reproductive organs while only one (CSP91) is found in male organs (Zhou et al., 2013). In *Adelphocoris suturalis* Jakovlev, *AsutCSP1* was expressed at higher levels in the male antennae than in the female antennae (Cui et al., 2017). The expression levels of two genes of *MmedNPC2* in males were both higher than expression levels in females (Zheng et al., 2018), suggesting that *MmedNPC2* in *M*. *mediator* may be involved in the perception of plant volatile organic compounds. The wide expressional profiling of those soluble chemical communication protein genes in different species suggests their functional diversity. They may play a chemosensory role in the olfactory system and may also play roles in other physiological processes, such as development, reproduction, and stress resistance (Bruno et al., 2018; Pelosi et al., 2018), lipid metabolism (Ishida et al., 2014; Pelosi et al., 2014), and cuticle synthesis (Foret et al., 2007). In this study, *AnilOBP9*, *AnilOBP26*, *AnilOBP83*, *AnilCSP5*, and *AnilNPC2* were expressed at higher levels in males than in females. These genes may encode proteins involved in sex‐specific behaviors, including selectively sensing and transporting sex pheromones released by females in the process of molecular recognition and searching for suitable mates. Higher levels of expression in males could be important for mating, reproduction, or other physiological processes. The expression level of *AnilCSP6* in females was significantly higher than in males, with high expression stimulated by β‐caryophyllene. This suggests that *AnilCSP6* is involved in host detection by *A*. *nilaparvatae* females.

## 5 CONCLUSION

We constructed the first high‐quality full‐length transcriptome database of *A*. *nilaparvatae*. The data obtained aid in understanding the complexity of *A*. *nilaparvatae's* transcriptome, as well as the sequence and functional annotation information of the complete reference genome. The molecular characteristics of soluble chemical communication proteins in *A*. *nilaparvatae* were discussed. Eight soluble chemical communication proteins were screened and identified, and their structures and phylogenetic relationships were determined. RT‐qPCR analysis suggested that *AnilCSP6* might be related to host detection by female wasps, but its specific functions need further study.

## CONFLICT OF INTEREST

Authors declare no conflict of interest.

## AUTHOR CONTRIBUTIONS

**Ying Ma:** Conceptualization (supporting); Data curation (lead); Formal analysis (supporting); Investigation (equal); Methodology (equal); Writing – original draft (lead); Writing – review & editing (supporting). **Tingfa Huang:** Data curation (equal); Formal analysis (lead); Investigation (lead); Methodology (equal). **Binjie Tang:** Project administration (supporting). **Bingyang Wang:** Conceptualization (supporting); Funding acquisition (supporting); Software (supporting). **Liyang Wang:** Visualization (lead). **Jianbai Liu:** Conceptualization (equal); Formal analysis (equal); Methodology (equal); Writing – review & editing (equal). **Qiang Zhou:** Conceptualization (lead); Data curation (equal); Funding acquisition (lead); Methodology (equal); Validation (equal); Writing – original draft (lead).

## Supporting information

Fig S1

Table S1‐S2

## Data Availability

Transcriptome sequences are available in NCBI accessions SRX11519542 and SRX11519541 (BioProject ID: PRJNA748197). All [Link], [Link] are available in Zenodo open data repository (https://doi.org/10.5061/dryad.sn02v6x5w).
