# Supplementary figures and images for: Transcriptome analysis and molecular characterization of soluble chemical communication proteins in the parasitoid wasp Anagrus nilaparvatae (Hymenoptera: Mymaridae)
Source: Ecol Evol. 2022 Mar 1;12(3):e8661. doi: 10.1002/ece3.8661 (PMC8888258; doi:10.1002/ece3.8661)

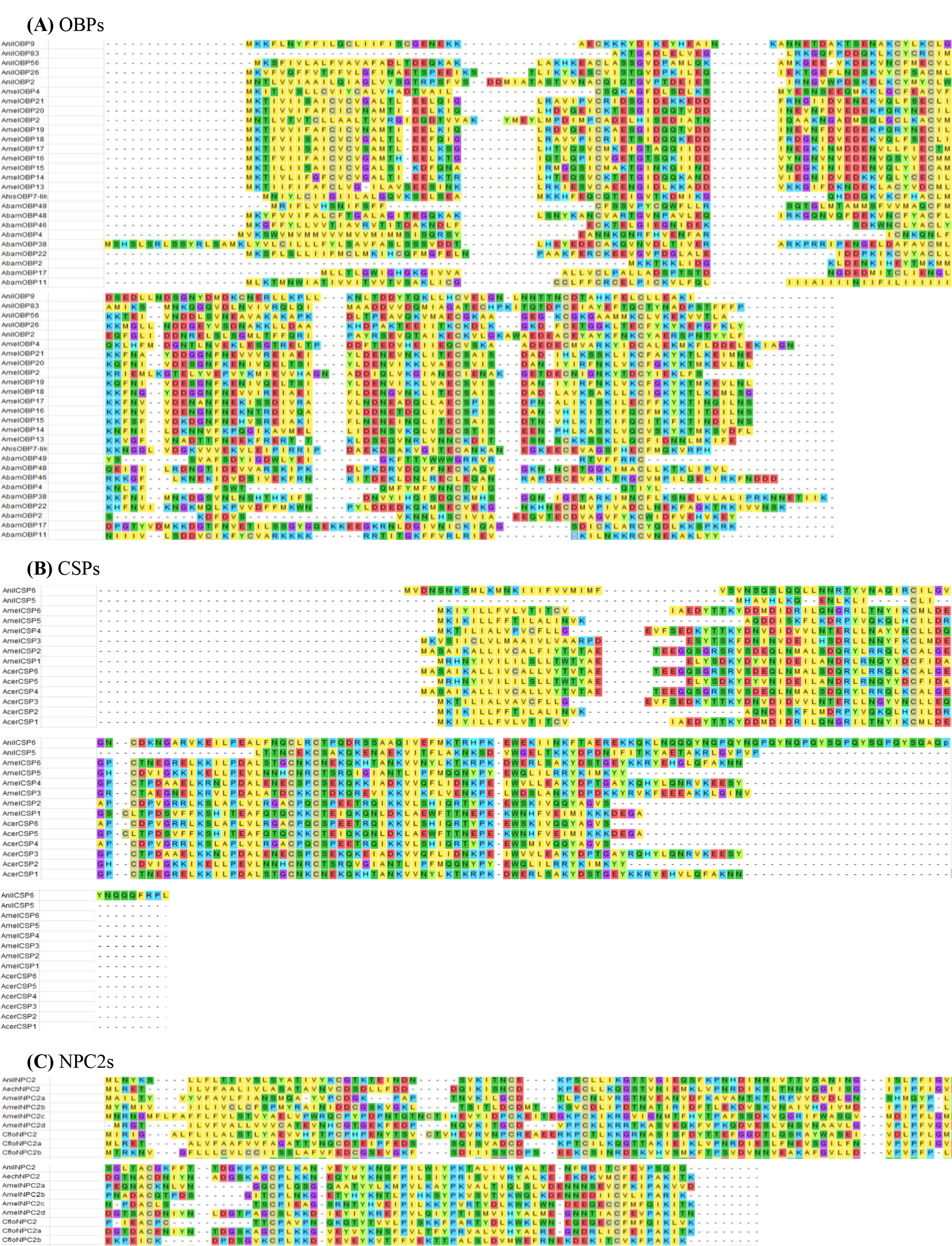

Supplement: Supplementary file 1 — Fig S1 [file ECE3-12-e8661-s002.jpg]
