# Supplementary material for: Transcriptome analysis and molecular characterization of soluble chemical communication proteins in the parasitoid wasp Anagrus nilaparvatae (Hymenoptera: Mymaridae)
Source: Ecol Evol. 2022 Mar 1;12(3):e8661. doi: 10.1002/ece3.8661 (PMC8888258; doi:10.1002/ece3.8661)
Supplement: Supplementary file 2 — Table S1‐S2 [file ECE3-12-e8661-s001.docx]

Supplementary Table 1 RT-qPCR primers for olfactory binding protein genes of *Anagrus nilaparvatae*

| Gene | Amplicon length (bp) | Primer name | Primer sequences (5’-3’) |
| --- | --- | --- | --- |
| AnilOBP2 | 94 | qOBP2-F  qOBP2-R | GGGCGTTCCTACAGATGAAA  AACATGCCGCTTATCAGCTC |
| AnilOBP9 | 92 | qOBP9-F  qOBP9-R | CATCAGCTGTGGAGAAAATGAG  TCGCTTGTCTTTGCATCTGT |
| AnilOBP26 | 96 | qOBP26-F  qOBP26-R | TACTGTTTCAGCGCCTGCAT  TTAGCTGGATCGTGTTTTGC |
| AnilOBP56 | 91 | qOBP56-F  qOBP56-R | CTTTTCGTTGCTGTCGCTTT  GCTGGGTCAACACCTGAACT |
| AnilOBP83 | 98 | qOBP83-F  qOBP83-R | AAATAATGGCAGCCGATGAC  TTCGTACGCGATCTCACAAG |
| AnilCSP5 | 88 | qCSP5-F  qCSP5-R | TGCAAAACAAAAAGAAAATGC  GGAACTCCAAGCCTTTTAGC |
| AnilCSP6 | 90 | qCSP6-F  qCSP6-R | AAATGGAGCACGCGTAAAAG  CCATTCTTTAGGGTGCCTTG |
| AnilNPC2 | 91 | qNPC2-F  qNPC2-R | TGATGGAAAACCTGCACCAT  TTTTCTGTCAGTGCCCAATGT |

Supplementary Table 2 Statistical results of Nanopore sequencing data and data corrected by Illumina

| Data | Total transcript bases | Tanscript number | Max length | Average transcript length | N50 |
| --- | --- | --- | --- | --- | --- |
| Nanopore sequencing data | 163.54 Mb | 224251 | 11203 | 729.27 | 998 |
| Data corrected by Illumina | 163.59 Mb | 224251 | 11225 | 729.49 | 998 |
